# Epigallocatechin gallate and curcumin inhibit Bcl-2: a pharmacophore and docking based approach against cancer

**DOI:** 10.1186/s13058-024-01868-9

**Published:** 2024-07-08

**Authors:** Noor Bahadar, Sher Bahadar, Abdul Sajid, Muqeet Wahid, Ghadir Ali, Abdullah Alghamdi, Hakeem Zada, Tamreez Khan, Shafqat Ullah, Qingjia Sun

**Affiliations:** 1 Department of Otorhinolaryngology, Head and Neck Surgery, The China-Japan Union Hospital of Jilin University, Xiantai Street 126, 130033, Changchun, Jilin China; 2 Key Laboratory of Molecular Epigenetics of the Ministry of Education (MOE), School of Life Sciences, Northeast Normal University, Renmin Street, Changchun, Jilin 130024 China; 3 College of Veterinary Sciences and Animal Husbandry, Abdul Wali Khan University Mardan, Mardan, Khyber Pakhtunkhwa Pakistan; 4 Department of Pharmacy, Bahauddin Zakariya University, Multan, 60800 Pakistan; 5 Department of Life Sciences, School of Science, University of Management and Technology (UMT), Lahore, 54700 Pakistan; 6 Department of Medical Laboratory, College of Applied Medical Sciences, Prince Sattam Bin Abdulaziz University, Al-Kharj, 11942 Saudi Arabia; 7 Mubarak Diagnostic Laboratory and Research Center, Peshawar, Pakistan

**Keywords:** Bcl-2, EGCG, Curcumin, Pharmacophore modeling, Molecular docking

## Abstract

The protein Bcl-2, well-known for its anti-apoptotic properties, has been implicated in cancer pathogenesis. Identifying the primary gene responsible for promoting improved cell survival and development has provided compelling evidence for preventing cellular death in the progression of malignancies. Numerous research studies have provided evidence that the abundance of Bcl-2 is higher in malignant cells, suggesting that suppressing Bcl-2 expression could be a viable therapeutic approach for cancer treatment. In this study, we acquired a compound collection using a database that includes constituents from Traditional Chinese Medicine (TCM). Initially, we established a pharmacophore model and utilized it to search the TCM database for potential compounds. Compounds with a fitness score exceeding 0.75 were selected for further analysis. The Absorption, Distribution, Metabolism, Excretion, and Toxicity (ADMET) analysis identified six compounds with favorable therapeutic characteristics. The compounds that successfully passed the initial screening process based on the pharmacodynamic model were subjected to further evaluation. Extra-precision (XP) docking was employed to identify the compounds with the most favorable XP docking scores. Further analysis using the Molecular Mechanics Generalized Born Surface Area (MM-GBSA) method to calculate the overall free binding energy. The binding energy between the prospective ligand molecule and the target protein Bcl-2 was assessed by a 100 ns molecular dynamics simulation for curcumin and Epigallocatechin gallate (EGCG). The findings of this investigation demonstrate the identification of a molecular structure that effectively inhibits the functionality of the Bcl-2 when bound to the ligand EGCG. Consequently, this finding presents a novel avenue for the development of pharmaceuticals capable of effectively addressing both inflammatory and tumorous conditions.

**Supplementary Information:**

The online version contains supplementary material available at 10.1186/s13058-024-01868-9.

## Introduction

The World Health Organization (WHO) reports that the worldwide prevalence of cancer affects around 10 million individuals. Based on projections from the WHO, the disease is anticipated to result in more than 27 million cases of illness and 17.5 million deaths by the year 2050 [1]. Cancer cells exhibit uncontrolled proliferation and are not influenced by growth signals. They can evade apoptosis, sustain angiogenesis, invade surrounding tissues, and metastasize, making metastatic disease a significant contributor to cancer-related deaths [2, 3]. The mitigation and prophylaxis of cancer can be accomplished by abstaining from risk factors and adhering to evidence-based preventive strategies. The management of cancer involves the application of chemotherapy and radiation therapy. Therapeutic interventions and pharmacological agents can potentially elicit non-specific toxicity in normal cells, hence resulting in drug resistance due to adverse effects [4]. It is imperative to develop pharmaceuticals derived from botanical sources that can undergo rigorous scientific assessment to determine their effectiveness, while prioritizing minimal adverse outcomes and a robust safety profile [5, 6]. Prior research has demonstrated the inhibitory properties of plant polyphenols on the growth of both chemically-induced and spontaneously occurring tumors. These findings suggest the necessity for additional inquiry in this area. Polyphenols can be employed alone or in combination to produce medications that efficiently target a wide range of metabolic processes. Polyphenols have anticancer characteristics and can be used with radiotherapy and chemotherapy to augment their effectiveness [7].

Historically, herbal remedies and therapies have been essential in treating and preventing various illnesses, including cancer. Phytochemicals greatly influence the development of cutting-edge cancer treatments and naturally occurring plant compounds. Phytochemicals called polyphenols are found naturally in many commonly consumed foods, including fruits, vegetables, tea, spices, nuts, dark chocolate, cereals, and wine. Based on their chemical composition, the approximately 8,000 distinct varieties of polyphenols can be further classified into multiple categories [8, 9]. These substances exhibit various structural variations but can be identified by one or more phenol rings. Polyphenols have several qualities that contribute to their potential as anticancer agents [10, 11]. Antioxidant, anti-inflammatory, antiproliferative, neuroprotective, and other traits fall under this category [12, 13].

Bcl-2, a known anti-apoptotic agent, has been associated with the advancement of cancer [14]. The Bcl-2 protein family regulates the intrinsic apoptosis pathway, forming a three-dimensional regulatory cassette. The chromosomal translocation may have identified oncoprotein Bcl-2, which differentiates human follicular lymphoma [15]. Its mechanism involves the inhibition of apoptosis. Its closely related homologues Bcl-xL, Bcl-w, Mcl-1, A1, and Bcl-B (in humans) also possess this anti-apoptotic action [16]. However, there are two distinct subgroups of apoptosis promoters. The first group is Bak, Bax, and the less researched Bok. These three proteins are similar to Bcl-2, especially in the three ‘BH’ (Bcl-2 homology) domains. Only the BH3 domain is shared by the second group, which includes Bim, Bad, Bid, Bik, Bmf, Puma, Noxa, and Hrk. The ‘BH3-only’ proteins insert amphibious α-helix domains into hydrophobic cognate pro-biotics in response to stress signals. These components work together to prepare the cell for programmed cell death, or apoptosis [17]. However, Bax and/or Bak proteins must be activated for apoptosis to initiate the intrinsic pathway. Once activated, these proteins aggregate in many inner membranes, including the outer membrane of mitochondria, disrupting the structural integrity of the membrane.

Identifying the initial gene that facilitated enhanced cell survival and growth rather than heightened proliferation has furnished compelling evidence underscoring the criticality of evading cellular demise in the progression of tumors [18, 19]. The inhibition of apoptosis occurs by inhibiting the cytochrome C release from mitochondria, thus preventing the caspases that trigger apoptosis from activation [20]. The upregulation of Bcl-2 has been associated with many advancements in cancer research. Neoplasms with limited differentiation and advanced stages have been linked to the overexpression of Bcl-2. The significance of Bcl-2 in angiogenesis and cancer development has been extensively established [21, 22]. Apoptosis represents the prevailing mechanism of cellular demise in response to tumor therapy [23]. Chemotherapeutic medications may enhance the induction of apoptosis through the specific targeting of Bcl-2. Bcl-2 inhibitors have demonstrated considerable potential as a therapeutic intervention for malignancies, with favorable outcomes both as monotherapy and in conjunction with other pharmacological agents across a wide range of malignancies [24].

This study examines the increasing importance of polyphenols in breast cancer against Bcl-2 protein in computational studies. The primary objective of this study is to explore the potential anticancer effects of many extensively studied polyphenols, including quercetin, kaempferol, gallic acid, and epigallocatechin gallate.

## Materials and methods

### Ligands

The 3-D conformers of 50 polyphenol ligands were retrieved from PubChem in sdf format. This study used TCMSP, a database of systems pharmacology for drug discovery from herbal medicines [25]. Maestro Ligprep module was used to optimize the geometry and energy settings of traditional Chinese medicine compounds. The ligPrep panel is a tool used to set up preparation calculations and start the configuration preparation for that. We aimed to generate 3D structures corresponding to the input 2D framework and the low-energy isomers that Glide and other programs needed. Using Epic, we obtained possible ionized states with a pH of 7.0 ± 2.0 and formed required tautomers, expect to get up to 32 stereoisomers per ligand, define chirality from available 3D coordinates, and produce rings with lower energy. Finally, the structure and energy of the compounds were minimized with the help of the OPLS3e force field.

### Ligand-based pharmacophore modeling and 3D QSAR study

The phase module of Schrodinger was used to construct the pharmacophore of a total of polyphenols with anticancer characteristics. Based on their pIC50 values, a total of active ligands was selected and subsequently divided into active and inactive groups. Following that, a pharmacophore model was built. The top five pharmacophore hypotheses with the highest survival scores were picked for additional study. This study used a pharmacophore matching tolerance of 2, 3D QSAR test, and a detailed analysis of the scoring functions. The ligands were minimized using a gradient-convergent optimal technique, and the ligands were later aligned by employing a flexible ligand alignment [26]. The ligands were randomly partitioned into training and test sets during validation. To construct the Quantitative Structure-Activity Relationship (QSAR) model, a training set comprising 70% of the data was retained.

### Pharmacophore modeling validations

The QSAR model was evaluated using a Partial Least Squares (PLS) factor 4. In the analysis, a grid space of size one was utilized. As a component of the validation procedure, the predicted activity of the compounds was assessed. The statistical measures used for evaluation were the squared correlation coefficient (R^2^) and the variance ratio (F). The pharmacophore model was validated using external test chemicals. Contour plots were used to determine the specific spatial locations within the structure with pharmacophoric requirements [26].

### ADMET

The Qikprop module of Maestro was used to assess the absorption, distribution, metabolism, excretion, and toxicity properties of the polyphenols obtained from the Chinese Traditional Medicine database. Additionally, the ADMET and drug-likeness parameters were also assessed using SWISS ADME (http://www.swissadme.ch) and ADMET Lab 2.0 (https://admetmesh.scbdd.com/) [27].

### Molecular docking

The molecular docking was conducted in Maestro [13]. The prime tool was used to fill structural gaps, and the Epik tool was employed to protonate het-groups at a pH of 7.0 ± 2.0. PROPKA optimized hydrogen bond arrangements at pH 7.0, and OPLS3e minimized constraint energy. A receptor grid box was generated using the Sitemap module, which determined the protein binding pocket coordinates and cubic grid boxes. The molecular docking process was carried out by employing prepared ligands, proteins, and receptor grid files. A molecular docking optimization was performed, extra-precision docking, and the Epik tool was used to carry out docking score penalties.

### MM-GBSA

The binding energies of Glide ligand-protein complexes were calculated using the MM-GBSA method. As stated in earlier literature, the predicted inhibition constant (Ki) was determined [28].

$${\rm{\Delta G = - RT}}\left( {{\rm{lnKi}}} \right){\rm{ }}\,{\rm{or}}\,\,{\rm{ Ki = e}}\left( {{\rm{ - \Delta G/RT}}} \right)$$

∆G is the ligand’s binding free energy, R is the gas constant (cal.mol^− 1^. K^− 1^), and T is room temperature (298 K).

### MDS

GROMACS 2022.3 (http://www.gromacs.org), a widely utilized molecular dynamics simulation software program, assessed the stability of the protein-ligand binding complex in the docked state. Based on molecular docking, molecular dynamics (MD) simulations were employed. The protein’s topology was generated utilizing the GROMACS software package and the CHARMM36 force field, whereas the topology for the ligand was generated using the Swiss-param server (https://www.swissparam.ch/). Subsequently, the protein-ligand complexes were immersed in a TIP3P water solution for solvation. The ligand-protein complex dissociation was achieved by introducing sodium (Na^+^) and chloride (Cl^−^) ions at a concentration of 0.15 mM into a cubic box with dimensions of 1 nm on each side. A notable decrease in the system’s energy consumption was achieved by employing a steep-descent methodology and executing a total of 50,000 iterations. The NVT ensemble, commonly called the canonical ensemble, and the NPT ensemble, also known as the isothermal-isobaric ensemble, were employed for a 200 picosecond ensemble equilibration. The temperature was reduced to 310 K using a modified Berendsen thermostat, while the Berendsen technique was employed to maintain the pressure at 1 atm. The electrostatic and van der Waals interactions at a 1 nm threshold were determined using the particle-mesh-Ewald and van der Waals approaches. Following a simulation of the system for ten nanoseconds, utilizing a time step of 2 fs, the subsequent trajectory was recorded at intervals of 10 picoseconds. Through the examination of the simulated trajectory data, it became feasible to quantify many parameters, namely the root mean square deviation (RMSD), fluctuation (RMSF), the radius of gyration (Rg), the solvent-accessible surface area (SASA), and the hydrogen-bond interactions [28].

#### Binding energies

Calculating free binding energies between proteins and their ligands is necessary to study reciprocal recognition and binding. The free energy of protein receptors reflects the strength of their interaction with ligands, so the positive and negative values of the free energy represent the possibility of interactions. The MM-PBSA approach was used to determine the binding free energy of a ligand-protein complex. In order to calculate the free binding energy, the gmx_MMPBSA module of GROMACS was used [25]. The last 100 frames of trajectory was used for analysis.

#### Entropy calculation, Protein residue energy calculation

In order to calculate the Entropy calculation, Protein Residue Energy calculation, the gmx_MMPBSA module of GROMACS was used.

#### Principal component analysis, and free energy landscape

In high-dimensional data, principal component analysis (PCA) is frequently used to find patterns [28]. This method can explain these changes by demonstrating concerted atomic displacements in MD trajectories and significant conformational changes between structures. This coordinated movement of atoms and conformational changes determines the structure and function of proteins. The data covariance matrix C is diagonalized to determine the primary components of the MD simulation trajectory. A principal component analysis was conducted using the GROMACS tools gmx_covar and gmx_anaeig.

GROMACS tool gmx sham was used to compute free energy landscapes (FEL). The following equation was used to calculate FEL:

$${\rm{\Delta G }}\left( {{\rm{PC1, PC2}}} \right){\rm{ }}\,{\rm{ = }}\,{\rm{ - KBTlnP }}\left( {{\rm{PC1, PC2}}} \right)$$

PC1 and PC2 represent the reaction coordinates, KB represents the Boltzmann constant, and P (PC1, PC2) represents the probability distribution. OriginPro software was used to plot FEL.

### Softwares

The process of molecular docking was conducted with Maestro v11.8 (Schrodinger suite 2018-4), Life Science: Maestro - Schrödinger (schrodinger.com). Molecular dynamics simulation was performed using the GROMACS 2022 software package (https://www.gromacs.org documentation).

## Results

### Ligand-based pharmacophore model

**Fig. 1:**
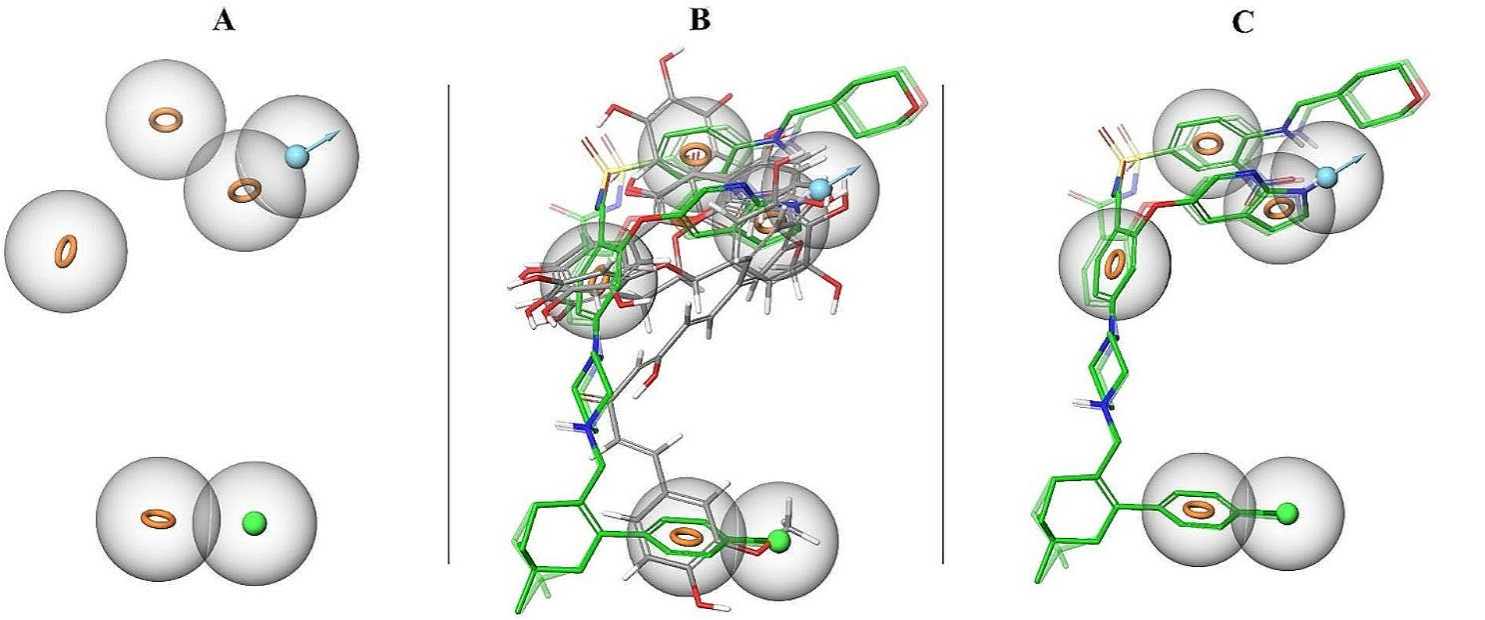
(**A**) Hypothesis (AAHNR): A: Acceptor (green), H: Hydrogen donor (blue), R: aromatic ring (orange rings). (**B**) Matched compounds fitted in hypothesis: Shows the compounds that fit the pharmacophore hypothesis. (**C**) Reference ligand fitted in hypothesis: Illustrates the reference ligand fitting the pharmacophore hypothesis

The concept of pharmacophore encompasses the essential physical and chemical attributes, as well as the spatial organization, that are required to identify ligands by biomacromolecules. The acquisition of polyphenols with specific targets or similar properties can be facilitated by utilizing the screening chemical databases alongside a pharmacophore model. Pharmacophore models could be classified into two primary classifications: structure-based models and ligand-based models. The present investigation entailed developing a series of three-dimensional pharmacophore models for Bcl-2 by utilizing established inhibitors. The examination of the shared features of biological activity involved the alignment of these inhibitory medicines. The optimized pharmacophore model (AAHNR) includes five distinct features: an aromatic ring, two hydrogen bond acceptors, a hydrogen bond donor, and a negatively charged ion core. This configuration is illustrated in Fig. 1. Table 1 shows that curcumin fits the model with a score of 1.18, compared to the reference ligand, which has a fitness score of 1.26. All other compounds have fitness scores below 1.

**Table 1:** Pharmacophore modeling of matched compounds

Matched Compound	Matched sites	Ligand Sites	Aligned Score	Vector Score	Volume Score	Fitness
Curcumin	4	D(8) H(11) R(12) R(-) R(-) R(13)	0.70	0.75	0.18	1.18
Epigallocatechin Gallate	4	D(15) H(-) R(21) R(20) R(22) R(-)	0.82	0.50	0.22	0.91
Kaempferol	4	D(9) H(-) R(12) R(13) R(11) R(-)	1.34	0.51	0.26	0.69
Myricetin	4	D(11) H(-) R(16) R(17) R(15) R(-)	1.34	0.31	0.26	0.50
Reference ligand	4	D(15) H(11) R(21) R(-) R(22) R(13)	0.63	0.67	0.11	1.26

D: hydrogen bond donor, H: Hydrophobic bond, and R: aromatic ring

### ADMET

The study utilized computational tools such as Qikprop, SWISS ADME, and ADMET Lab 2.0 to generate predictions for various physiochemical, medicinal chemistry, and ADMET parameters. These predictions were developed for polyphenols identified using a structure-based pharmacophore model (Table 2). Every chemical exhibited a distinct and noteworthy attribute. As a result, the application of ADMET analysis has enabled the discovery of ligands that exhibit pharmacokinetic characteristics that meet acceptable thresholds. The pharmacokinetic characteristics of all drugs were determined to be highly favorable, with no side effects determined in computational studies. However, further in vitro and in vivo studies are needed to determine the toxic effects. Moreover, a widely held perception existed that the prospects for therapeutic applications were promising.

**Table 2:** ADMET analysis of selected polyphenols

Parameters	Myricetin	Epigallocatechin gallate	Curcumin	Kaempferol
LogS	-3.665	-3.805	-3.921	-3.624
LogD	0.9	1.058	2.82	2.233
LogP	1.747	1.862	2.742	2.656
Pgp-Inhibitor	0.004	0.019	0.284	0.004
Pgp-Substrate	0.006	0	0.014	0.011
HIA	0.035	0.914	0.06	0.008
F(20%)	0.977	0.999	0.011	0.856
F(30%)	0.999	1	0.171	0.993
Caco-2	-5.653	-6.608	-4.834	-4.974
MDCK	6.38E-06	4.56E-06	1.63E-05	9.07E-06
BBB	0.006	0.006	0.103	0.009
PPB	92.77%	87.88%	99.80%	97.86%
VDss	0.633	0.521	0.369	0.522
Fu	10.35%	8.26%	1.05%	4.41%
CYP1A2-Inhibitor	0.846	0.239	0.593	0.972
CYP1A2-Substrate	0.108	0.071	0.758	0.11
CYP2C19-Inhibitor	0.027	0.026	0.287	0.181
CYP2C19-Substrate	0.037	0.035	0.12	0.046
CYP2C9-Inhibitor	0.574	0.566	0.661	0.653
CYP2C9-Substrate	0.32	0.204	0.906	0.867
CYP2D6-Inhibitor	0.098	0.013	0.037	0.722
CYP2D6-Substrate	0.169	0.185	0.895	0.283
CYP3A4-Inhibitor	0.187	0.098	0.674	0.697
CYP3A4-Substrate	0.025	0.106	0.517	0.08
CL	7.716	16.556	13.839	6.868
T12	0.945	0.928	0.948	0.905
hERG	0.145	0.102	0.214	0.07
H-HT	0.099	0.235	0.475	0.098
DILI	0.982	0.905	0.895	0.979
Ames	0.482	0.153	0.234	0.672
ROA	0.022	0.032	0.896	0.156
FDAMDD	0.557	0.113	0.776	0.109
SkinSen	0.943	0.971	0.958	0.856
Carcinogenicity	0.028	0.023	0.706	0.097
EC	0.008	0.003	0.007	0.009
EI	0.93	0.933	0.792	0.929
Respiratory	0.065	0.02	0.951	0.09
BCF	0.939	0.974	0.8	0.986
IGC50	3.885	4.154	5.077	4.386
LC50	4.982	5.144	6.191	5.223
LC50DM	5.272	5.596	5.194	5.205
NR-AR	0.006	0.006	0.807	0.008
NR-AR-LBD	0.152	0.087	0.787	0.371
NR-AhR	0.968	0.782	0.596	0.967
NR-Aromatase	0.889	0.691	0.619	0.941
NR-ER	0.918	0.846	0.6	0.959
NR-ER-LBD	0.985	0.991	0.624	0.985
NR-PPAR-gamma	0.954	0.865	0.957	0.963
SR-ARE	0.769	0.561	0.891	0.873
SR-ATAD5	0.292	0.109	0.647	0.612
SR-HSE	0.535	0.941	0.884	0.557
SR-MMP	0.962	0.968	0.871	0.968
SR-p53	0.903	0.712	0.906	0.921
MW	318.04	458.08	368.13	286.05
Vol	291.557	425.17	381.036	273.977
Dense	1.091	1.077	0.966	1.044
nHA	8	11	6	6
nHD	6	8	2	4
TPSA	151.59	197.37	93.06	111.13
nRot	1	4	8	1
nRing	3	4	2	3
MaxRing	10	10	6	10
nHet	8	11	6	6
fChar	0	0	0	0
nRig	18	24	16	18
Flex	0.056	0.167	0.5	0.056
nStereo	0	2	0	0
NonGenotoxic_Carcinogenicity	0	0	1	0
LD50_oral	0	0	0	0
Genotoxic_Carcinogenicity_Mutagenicity	0	0	1	0
SureChEMBL	1	1	0	0
NonBiodegradable	1	1	1	1
Skin_Sensitization	8	9	8	4
Acute_Aquatic_Toxicity	0	0	2	0
Toxicophores	2	2	3	1
QED	0.371	0.212	0.548	0.546
Synth	2.733	3.74	2.426	2.375
Fsp3	0	0.136	0.143	0
MCE-18	20	87.48	14	18
Natural Product-likeness	1.698	1.65	0.722	1.546
Alarm_NMR	3	3	3	2
BMS	1	1	0	0
Chelating	2	1	2	1
PAINS	1	1	0	0
Lipinski	Accepted	Rejected	Accepted	Accepted
Pfizer	Accepted	Accepted	Accepted	Accepted
GSK	Accepted	Rejected	Accepted	Accepted
GoldenTriangle	Accepted	Accepted	Accepted	Accepted

### Binding pocket prediction

**Fig. 2:**
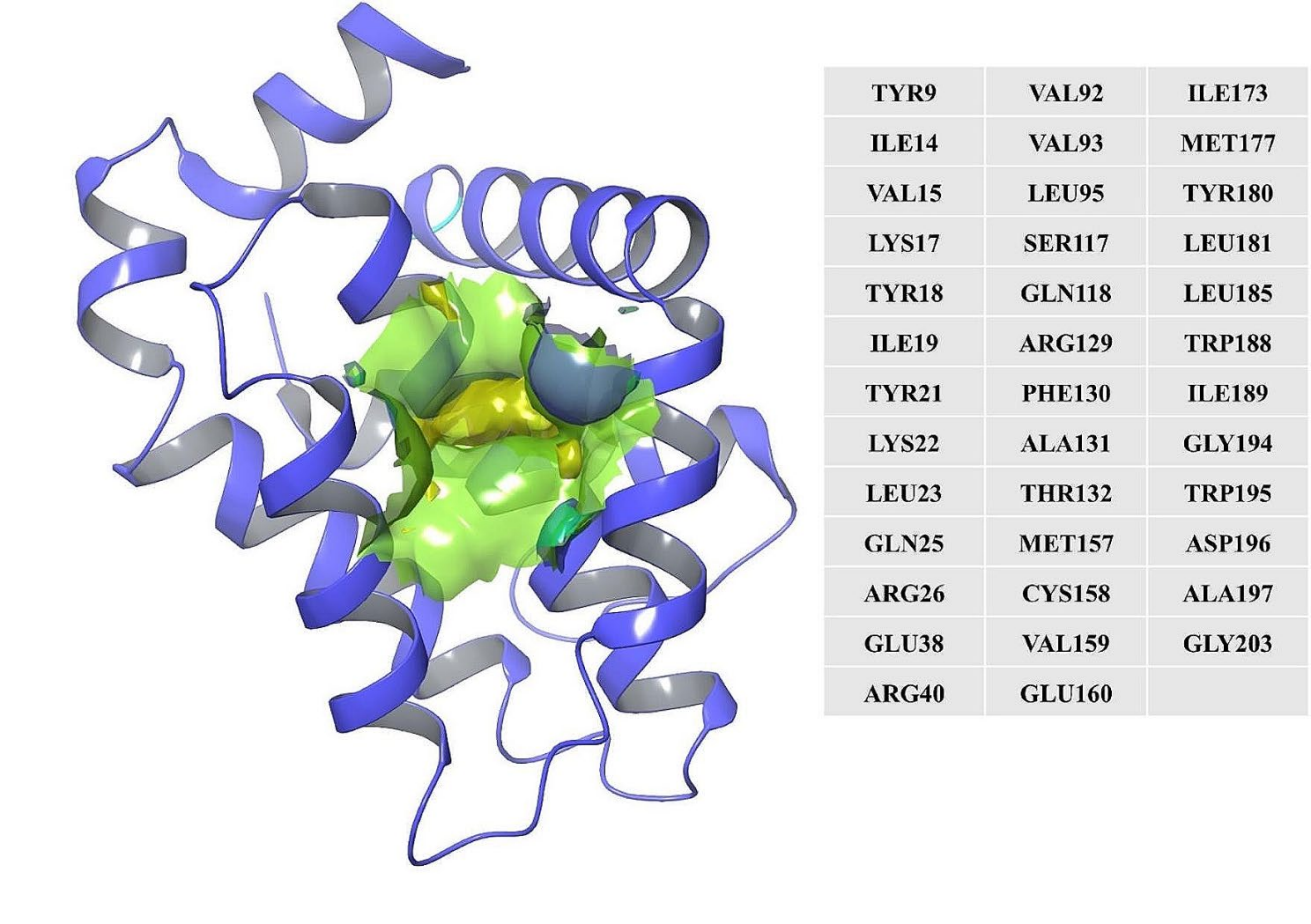
Binding site pocket with their residues of the predicted Bcl-2 protein retrieved from the Sitemap module

The binding site was identified from the Maestro Sitemap module. Figure 2 represents the binding site and list of residues of the binding pocket.

### Molecular docking

**Fig. 3:**
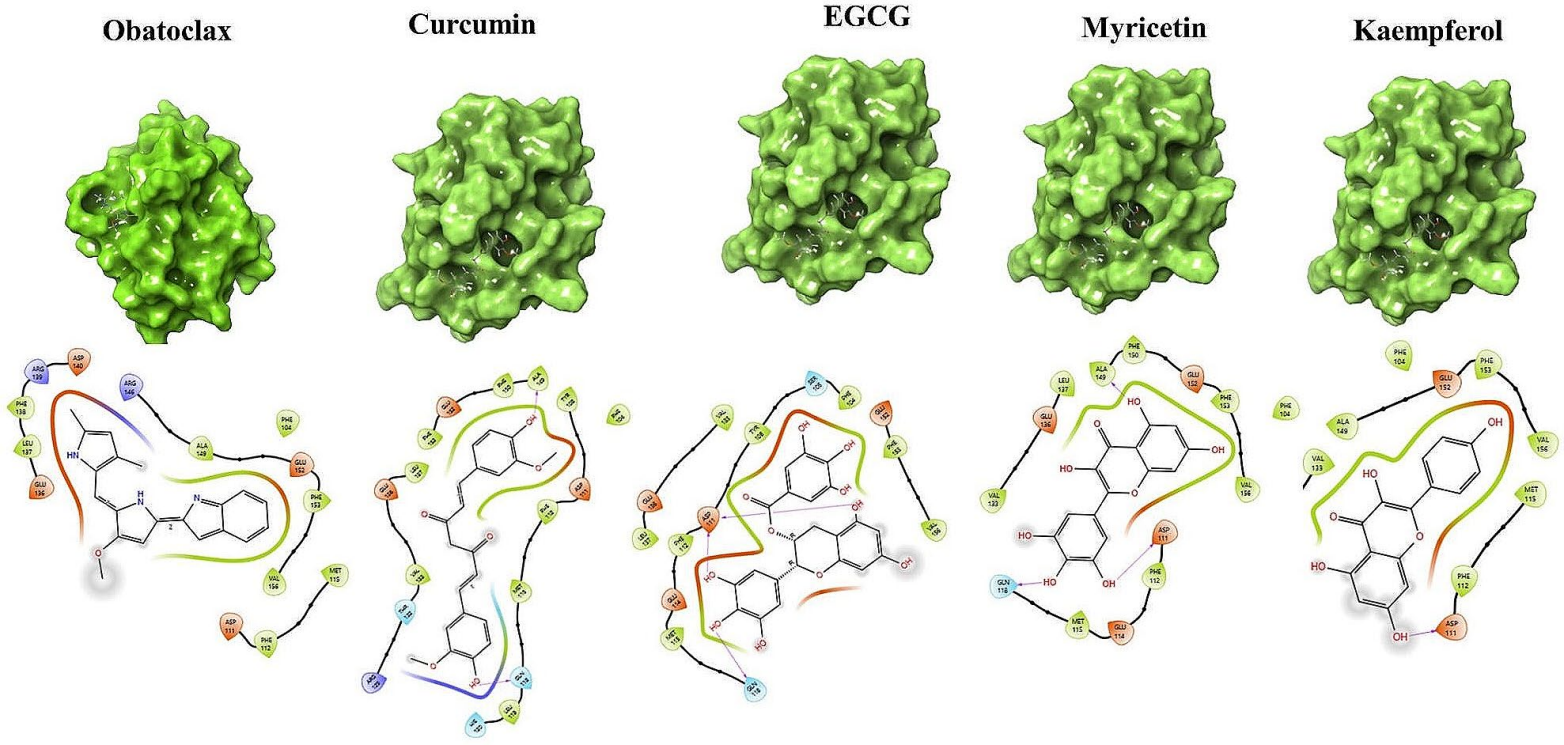
2D ligand-protein interaction between Bcl-2 and ligands: Obatoclax, Curcumin, Epigallocatechin gallate (EGCG), Myricetin, and Kaempferol

The application of molecular docking plays a crucial role in the drug design process, as it enables the identification of the bioactive conformation of small and medium-sized molecules at protein binding sites and the examination of interactions between protein ligands. The Glide module utilizes an approach that systematically investigates the orientation, conformation, and spatial disposition of docking ligands to analyze molecules. The first step in the process entails minimizing the search area by use of approximate positioning and evaluation. The energy optimization of the candidate posture is subsequently performed via the OPLS-AA non-bonding potential grid. As a result of energy minimization, it had − 5006.8 kcal/mol from non-minimized protein, which has zero energy. The selection and assessment of the optimal docking position is ultimately determined by applying a functional model that incorporates experiential knowledge and force field analysis. The current study focused on creating receptor grids using the Glide module, with coordinates X = -11.072, Y = 1.71, and Z = -17.085. The Maestro software was utilized to conduct molecular docking of the compounds with Bcl-2 to evaluate and analyze their binding affinity. The comparative study of four polyphenols targeting Bcl-2 is depicted in Table 1, including their relative docking and MMGBSA scores. The binding affinities of Myricetin, Epigallocatechin gallate, Curcumin, and Kaempferol were measured to be -29.79 kcal/mol, -34.83 kcal/mol, -56.02 kcal/mol and-39.22 kcal/mol respectively (Fig. 3; Table 3).

**Table 3:** Docked ligand-protein complex binding energies (kcal/mol) calculated with Prime MM–GBSA

Compounds	Docking score kcal/mol	Glide energy kcal/mol	∆G Bind kcal/mol	Log pKi	∆G Bind Coulomb kcal/mol	∆G Bind Covalent kcal/mol	∆G Bind Hbond kcal/mol	∆G Bind Lipo kcal/mol	∆G Bind Packing kcal/mol	∆G Bind Solv GB kcal/mol	∆G Bind vdW kcal/mol	Residues Interactions
Myricetin	-7.232	-36.589	-29.79	-9.71	-19.44	8.93	-2.09	-6.21	-0.89	22.23	-32.32	**Hydrogen Bond**: Ala149, Gln118, Asp111, Gln118, **Hydrophobic Bond**: Met115, Met115, Ala149, Met115
Epigallocatechin gallate	-6.971	-38.533	-34.83	-11.90	-17.97	3.88	-2.19	-15.58	-0.34	31.38	-34.01	**Hydrogen Bond**: Arg146, Arg146, Asp111, Gln118, Asp111, Ala149, Ala149, **Hydrophobic Bond**: Met115, Met115, Met115, Ala149, Leu137, Arg146
Curcumin	-5.929	-40.114	-56.02	-21.10	-16.52	6.14	-1.42	-23.58	-0.7	19.17	-39.11	**Hydrogen Bond**: Gln118, Leu119, Ala149, Arg129, Arg129 **Hydrophobic Bond** Phe104, Met115, Leu137, Ala149
Kaempferol	-5.914	-29.25	-39.22	-13.80	-6.89	1.93	-1.23	-14.11	-0.52	13.04	-31.43	**Hydrogen Bond**: Asp111, Leu137 **Hydrophobic Bond**: Met115, Met115, Ala149
Obatoclax	-5.644	-29.594	-42.21	-8.89	2.1	-0.27	-20.06	-0.17	-8.89	22.86	-37.77	**Hydrogen Bond**: Glu136, Leu137 **Hydrophobic Bond**: Leu137, Met115, Met115, Ala149,

### Molecular dynamic simulations

MD simulation is a broadly used method in examining protein-ligand complexes, primarily focused on understanding their stability. The molecular dynamics (MD) simulation showed an observable temporal progression of binding modes within protein-ligand complexes. Within the framework of typical MD simulations, the interactions of atoms and molecules are governed by a force field. During the simulation procedure, the computation of many parameters, such as the potential energy, the total energy, the temperature, and the pressure, of the protein-ligand combination was conducted.

**Fig. 4:**
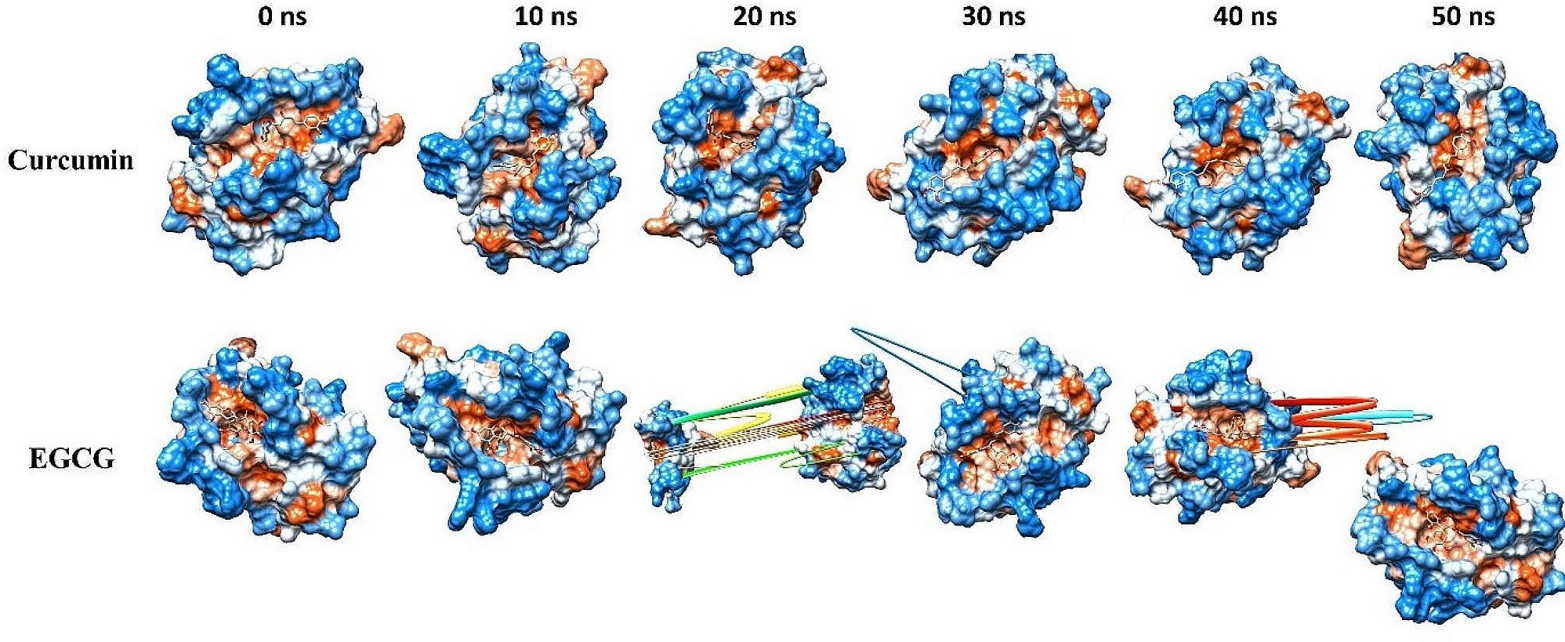
The comparison of protein conformational changes at different molecular dynamic allosteric states

**Fig. 5:**
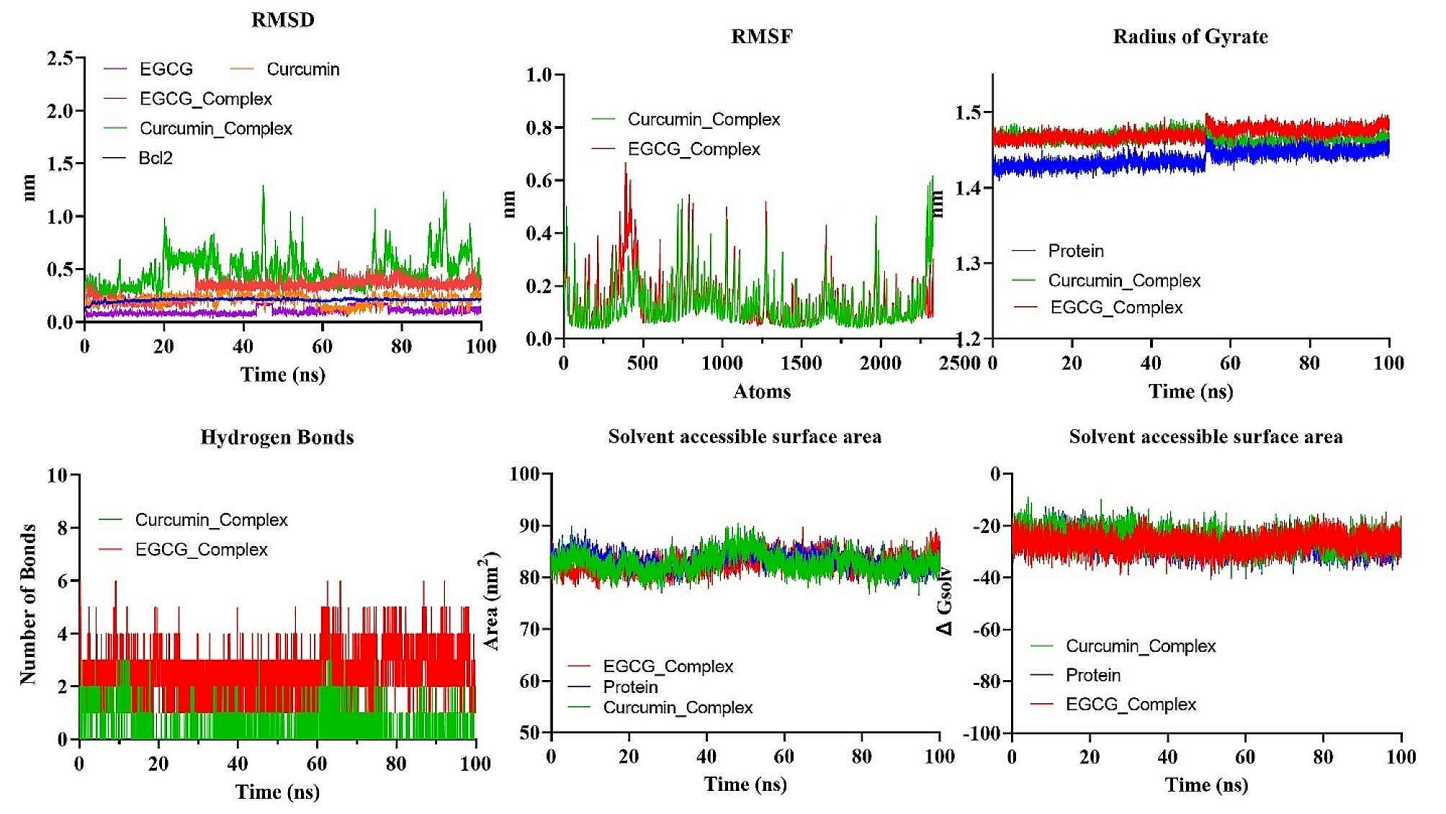
The analysis of MD simulations trajectory of docked complexes, i.e., Bcl-2 Curcumin and Bcl-2 EGCG for 100 ns. Blue: Bcl-2 Protein, Green : Curcumin Bcl-2 Complex, Red: EGCG Bcl-2 Complex

The evaluation of the stability of intricate chemical and protein systems, along with the stability of proteins subsequent to the introduction of small molecules and amino acid hydrophobicities, can be accomplished by quantifying molecular dynamics (MD) parameters such as RMSD, RMSF, Rg, and SASA. Based on Glide energy and ∆G binding energy, we selected the curcumin and Epigallocatechin gallate (EGCG), also known as epigallocatechin-3-gallate, for MD simulations. Both compounds showed the highest binding affinity with Bcl-2. Therefore, the selection of protein-ligand complexes, including Curcumin and EGCG, in conjunction with Bcl-2, was made to conduct MD simulations. Figure 4 shows the protein conformational changes at different dynamic allosteric states, and it was observed that curcumin had stability in pockets of protein Bcl-2, especially at 40 and 50 ns. But EGCG faced instability at 20, 30, and 40 ns. The results are illustrated in Fig. 5.

#### RMSD

The quantitative assessment of the stability of the protein-ligand system is performed employing the root-mean-square deviation (RMSD). RMSD of the protein backbone atoms in each complex exhibited a consistently low value during MD simulations (Fig. 5). The mean RMSD of the docked complexes Bcl-2-curcumin and Bcl-2-EGCG were determined to be 0.4778 ± 0.001541 nm and 0.3174 ± 0.0008145 nm, respectively.

#### RMSF

The analytical technique called Root Mean Square Fluctuation (RMSF) enables the detection of alterations in amino acid residues within a protein during a designated temporal interval. A higher RMSF value indicates substantial fluctuations in the residues, whereas a lesser RMSF value indicates reduced levels of volatility in the residues. The impact of curcumin and EGCG on the variability of individual protein residues was evaluated by determining the RMSF of backbone atoms for each residue within the protein complex. The mean RMSF values of the Bcl-2-curumin and Bcl-2-EGCG docked complexes were 0.1262 ± 0.001762 nm and 0.1408 ± 0.001990 nm, respectively (Fig. 5). No noticeable conformational changes were seen in the active sites, and the binding residues responsible for EGCG exhibited consistently stable activity.

#### Radius of gyration (rg)

The gyratory radius is a critical measure used to assess the level of compactness shown by protein structures. Proteins with a reduced gyratory radius exhibit a more compact conformation, indicating a higher degree of stability in their structural arrangement. The average Rg values for the docked complexes of Bcl-2 with curcumin and Bcl-2 with EGCG were determined to be 1.466 ± 0.00008576 nm and 1.472 ± 0.00009149 nm, respectively. The lack of substantial deviations in the trajectory of the radius of gyration indicates the absence of anomalous behavior (Fig. 5).

#### Solvent accessible surface area (SASA)

The SASA metric quantifies the degree of hydrophobicity a protein shows, whereby larger SASA values correspond to increased protein content and less temporal variability during simulation. Proteins can undergo structural modifications by adding small chemicals, which can induce substantial alterations in the solvent-accessible surface area (SASA). The complexes formed by the compound-protein interaction exhibited a predominantly stable behavior throughout the 100-nanosecond simulation. The average solvent-accessible surface areas (SASA) of Bcl-2-curcumin and Bcl-2-EGCG complexes were determined to be 82.80 ± 0.01915 nm2 and 82.82 ± 0.01517 nm2, respectively. (Fig. 5)

#### Hydrogen bond

The formation of a hydrogen bond considerably enhances a ligand’s capacity to interact with a protein and be acknowledged by the active site. During a 100 nanosecond MDS, the gmx_hbond module in GROMACS was employed to analyze the number, length, and angle of hydrogen bonds inside three protein-rutin complexes. The mean quantity of hydrogen bonds formed throughout a 100 nanosecond molecular dynamics simulation was determined to be 0.5098 ± 0.006317 for the Bcl-2-curumin complex, while the Bcl-2-EGCG docked complex exhibited an average of 2.495 ± 0.09108 hydrogen bonds (Fig. 5). The simulation demonstrated that the hydrogen bond interaction between the ligands and protein showed a state of sustained and enduring stability.

#### Binding energies

**Fig. 6:**
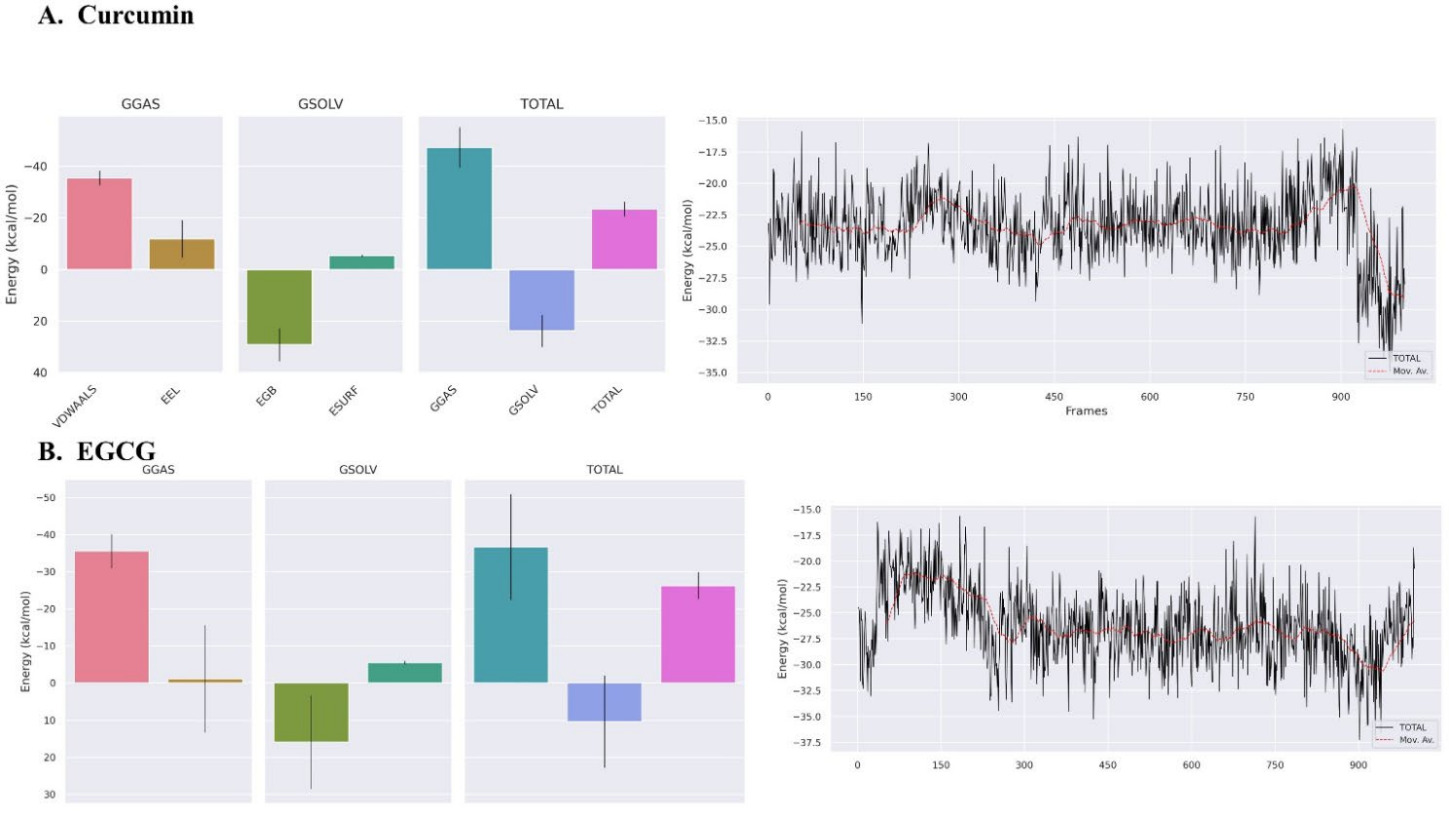
Total free energy components of binding (∆G_TOTAL_) and running average (red line) of binding energies over 1000 frames. (A) Binding energies of Curcumin (B) Binding energies of EGCG

The binding free energy of the complex system was calculated using the MM-PBSA method implemented in the gmx-MMPBSA tools. Negative values for the Gibbs free energy and enthalpy signify that the ligand binding process is exothermic and creates thermodynamically stable complexes (Fig. 6). The gmx MMPBSA test tool was utilized to calculate the binding free energy of the three most favorable compounds. The visualization capabilities provided by gmx_MMPBSA_ana were used to generate graphical representations and assess the energy components associated with the MM/GBSA methodology.

Figure 6 displays the energy values for the combined components (∆G_GAS_, ∆G_SOLV_, and ∆G_TOTAL_). The line graphs depicted the fluctuation in binding free energy and the corresponding running average fluctuation seen over the final 100 ns of the molecular dynamics simulation trajectory. The EGCG has a significantly elevated ∆G_TOTAL_ binding free energy of -26.25 ± 3.62 kcal/mol. In contrast, the curcumin, which had a binding free energy of -23.47 ± 2.89 kcal/mol, exhibits a comparable pattern. This study utilized a sample of 1000 frames extracted from the final 100 nanoseconds of the molecular dynamics (MD) simulation. The frames were assessed to ascertain the numerical value of ∆G_TOTAL_, which is provided in conjunction with them. The ∆G_TOTAL_ running average is visually represented by a dashed red line. The data reveals that EGCG exhibited an increasing trend in the running average of the ∆G_TOTAL_ parameter. The subsequent frames showed a gradual decline, ultimately reaching a steady state. The mean value of ∆G_TOTAL_ for the curcumin exhibited a notable reduction.

#### Entropy calculation

**Fig. 7:**
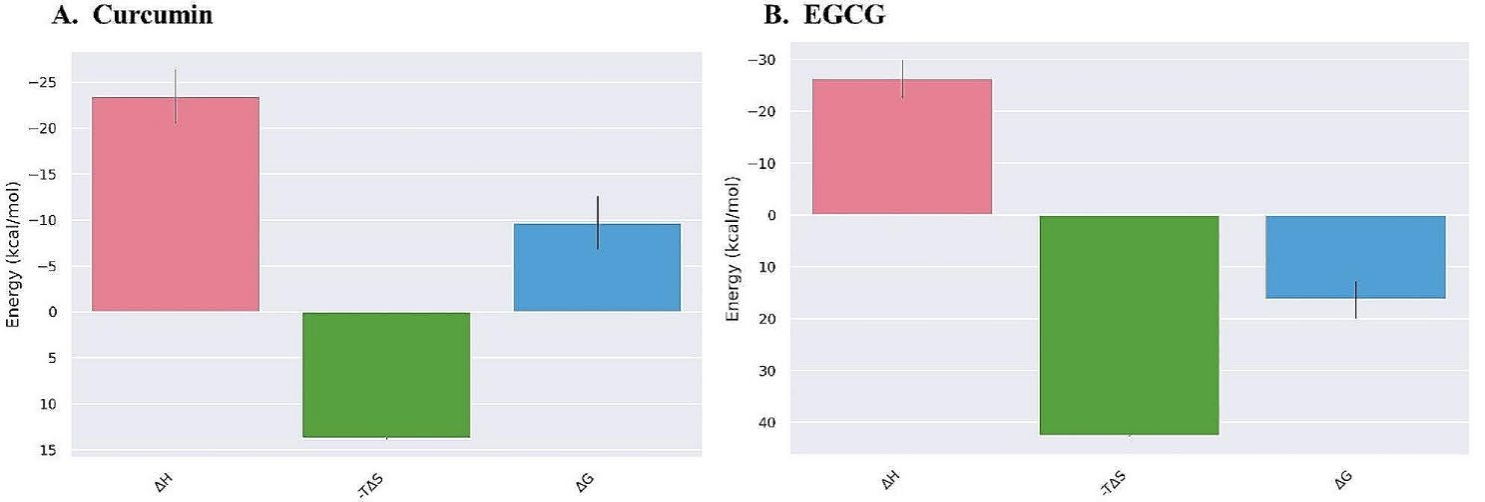
The enthalpy change (∆H), entropy (-T∆S), and binding free energy (∆G) of protein-ligand complexes were demonstrated based on MM/GBSA for curcumin and EGCG.

The binding free energies for the ligands were calculated by evaluating the enthalpy change (∆H) and the entropy contribution (T∆S) using the gmx MMPBSA test tool. Figure 7 presents entropy calculation, including the entropic factors enthalpy (∆H), entropy (-T∆S), and binding free energy (∆G). In general, the observed entropy changes in all three systems exhibited negative values, suggesting that the protein-ligand complex had lower entropy compared to the separate protein and ligand entities in their unbound states. The study revealed that the entropy factors for EGCG were determined to be -T∆S 42.58 kcal/mol, ∆H -26.25 ± 3.62 kcal/mol, and ∆G 16.33 ± 3.63 kcal/mol. In contrast, curcumin exhibited -T∆S of 13.77 ± 0.11 kcal/mol, ∆H of -23.47 ± 2.89 kcal/mol, and ∆G of -9.7 ± 2.9 kcal/mol. The existence of high entropy levels has an impact on the stability of the interaction between medical medicines and protein targets in a broader context.

#### Protein residue energy calculation

**Fig. 8:**
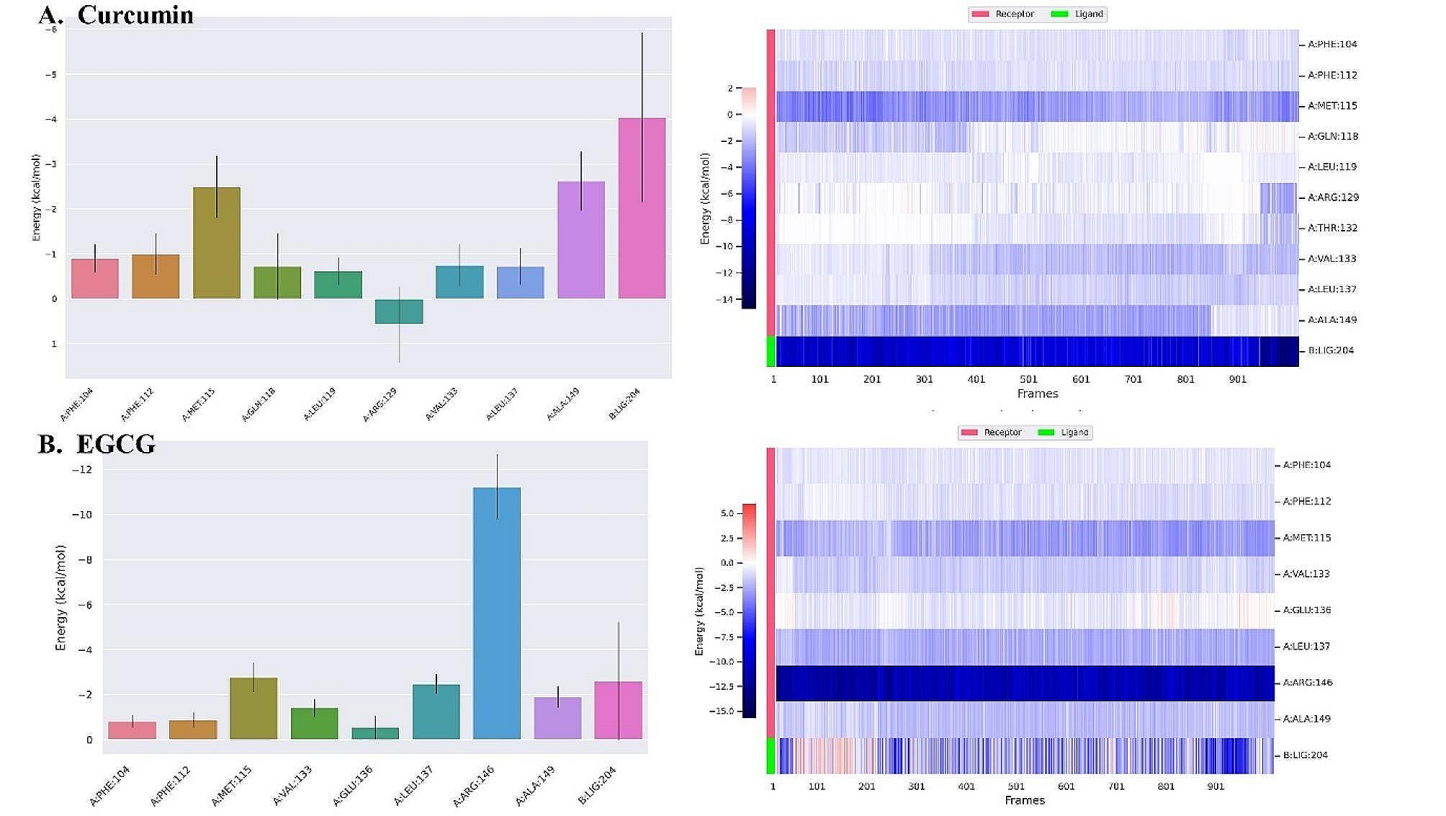
The Bindings energy of protein residues with ligands (**A**) Curcumin and (**B**) EGCG

Figure 8 presents binding energy calculations for protein residue in the protein-ligand complex over 1000 frames. In the Bcl-2 curcumin complex, MET 115 had prominent binding energy. In contrast, Bcl-2 EGCG complex Arg 146 had noticeable binding energy.

#### Principal component analysis and free energy landscape (FEL)

**Fig. 9:**
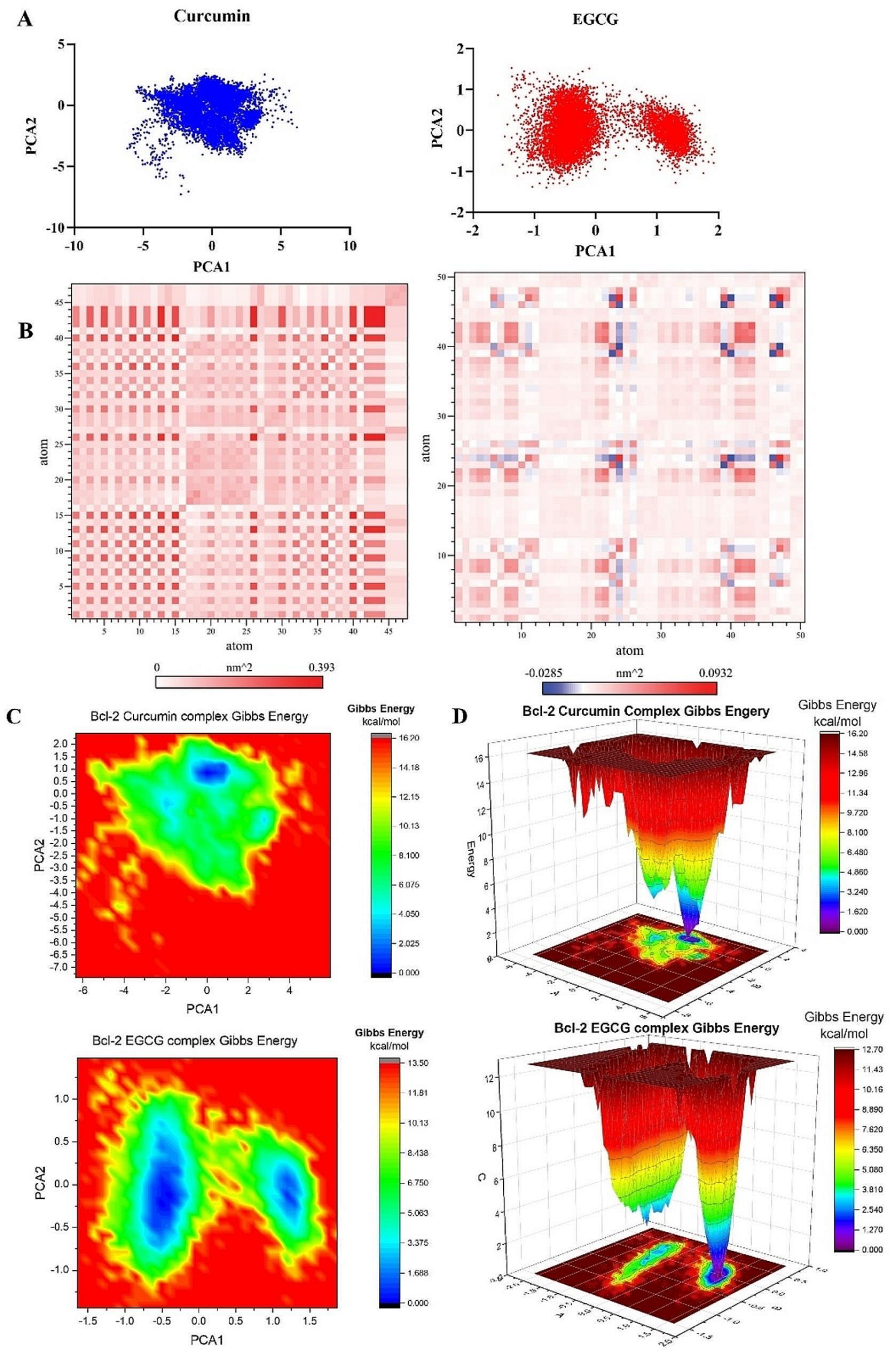
Principal component analysis (PCA), Covariance, and free energy landscape (FEL) of Bcl-2 Curcumin and Bcl-2 EGCG docked complexes for 100 ns. (**A**) 2D PCA plots (**B**) Covariance plots for residues of proteins. (**C**) 2D PCA-based energy plots. D.3D FEL of docked complexes

Principal component analysis (PCA) is a commonly employed methodology for reducing the dimensionality of extensive datasets to extract pertinent information. Principal component analysis (PCA) was used to investigate the mobility modes of docked complexes consisting of Bcl-2 and its ligands, specifically curcumin and EGCG. The docked complexes of Bcl-2 curcumin and Bcl-2 EGCG are depicted in Fig. 9A using principal component analysis (PCA) plots. The implementation of routine binding procedures resulted in the formation of robust complexes. A research investigation was conducted to examine the dynamic covariance matrix of docked protein complexes to identify residues associated with ligands curcumin and EGCG demonstrating anti-correlated motion. Figure 9B displays the covariance matrix of the complexes formed by Bcl-2 curcumin and Bcl-2 EGCG. The utilization of variations in color intensity serves as a visual representation of the correlation coefficient, a statistical measure that ranges from − 1 to 1.

Free energy landscape analysis investigated the low energy conformations in the test systems involving Bcl-2 curcumin and Bcl-2 EGCG. The generation of free energy graphs in three-dimensional (3D) and two-dimensional (2D) formats was accomplished by utilizing the first and second fundamental components. The free energy landscapes of Bcl-2 EGCG and Bcl-2 curcumin are depicted in Figs. 9C and 10D. The free energy landscape shows energetically favorable conformations as blue dots, whereas energetically unfavorable conformations are represented by red dots. The free energy landscape of the Bcl-2 EGCG complex exhibits a higher concentration of blue dots compared to the Bcl-2 curcumin complex, indicating that the interaction between rutin and the active site of Bcl-2 EGCG resulted in an energy reduction.

## Discussion

Currently, various approaches are employed in the therapeutic targeting of neuroblastoma cell lines by using natural compounds, such as flavonoids. Four principal therapeutic processes can be classified according to their mechanisms: cell proliferation inhibition, receptor-facilitated apoptosis, calpain-induced apoptosis, and activation of apoptosis via mitochondrial endoplasmic reticulum pathways [29, 30]. The initiation of apoptosis depends upon the relative quantities of mitochondrial protein subfamilies, namely Bcl-2 (including the anti-apoptotic Bcl-xL) and the pro-apoptotic Bcl-2 associated X-protein (Bax). It has been established that an increased Bax/Bcl-2 ratio is related to the initiation of cytochrome c release from the mitochondria [31, 32]. Consequently, the activation of caspases-9 and − 3, which initiate the process of programmed cell death known as apoptosis, takes place. The induction of poly (ADP-ribose) polymerase (PARP) fragmentation is also observed with an elevated Bax to Bcl-2 ratio. Numerous flavonoids, including apigenin, butein, DEDC, genistein, luteolin, EGC, EGCG, and quercetin, have been reported to elicit an overexpression of the Bax protein in malignant cells [33, 34]. Prior research has indicated that non-flavonoid polyphenols, including bisphenol-A (BPA), bisphenol-B (BPB), bisphenol-S (BPS), honokiol, and resveratrol, have had similar effects on Bax [35]. Several flavonoids, including apigenin, butein, carnosic acid (CA), DEDC, genistein, and rutin, have exhibited the ability to reduce the synthesis of Bcl-2 and Bcl-xL proteins.

Moreover, certain non-flavonoid polyphenols, such as BPA, BPB, BPS, curcumin, and resveratrol, have also demonstrated this capability. The phenomenon of a decrease in neuroblastoma (NB) is accountable for the liberation of cytochrome c and the fragmentation of PARP [36, 37]. Curcumin, EGC, and EGCG are polyphenolic compounds that have been shown to possess potent apoptotic properties through receptor-mediated mechanisms. The apoptotic processes that were identified included several noteworthy events, such as increased levels of TNF-α, activation of caspase-8, generation of tBid, association with the adaptor protein FADD, and oligomerization of Bax [38]. In receptor-mediated apoptosis, the Bax/Bcl-2 ratio plays a critical role, akin to previous research indicating that mitochondrial/endoplasmic reticulum pathways trigger apoptosis. The dependence of many receptor-mediated apoptotic pathways on mitochondrial apoptotic pathways demonstrates the considerable importance of mitochondria in the apoptosis of treated neuroblastoma cell lines. The pro-apoptotic protein Bax is crucial in initiating the mitochondrial apoptotic pathway and is generally recognized as a critical target in anticancer treatment [39]. Consequently, it was anticipated that the biomarkers Bax and Bcl-2 would exhibit a substantial presence.

Experiments have shown that curcumin has great opportunities for targeting in the field of cancer research. It is found that curcumin has a binding energy strength of about − 10 kcal/mol concerning both EGFR as well as NF-κB proteins [40]. This binding energy is similar to other compounds of curcumin, which are seen to have higher binding energy than curcumin molecules. It has shown a docking score of -10, which advocates its use in a medicinal setting. The binding affinity was determined to be 36 kcal/mol when interacting with ALDH1A1 and − 10. The calculated binding energies for molecule 4 were 62 kcal/mol when it was complexed with the target enzyme, GSK-3β. Encouragingly, several curcumin analogs have demonstrated enhanced inhibition of GSK-3β by having a higher scoring value of -10 for compound 37e. 91 kcal/mol. Some of the parameters obtained were a binding affinity of -7. 5 kcal/mol when engaging with the FGFR-1 receptor, depending on the type of bond it forms. This binding affinity is near that of other compounds like lapatinib, which has a binding affinity of -8. 5 kcal/mol with FGFR-1. From the ΔG binding calculated above, curcumin and LAP showed comparable or higher binding free energy than two known inhibitors of MMP-3, PBSA, and MPPT [41, 42]. These studies demonstrate that curcumin’s binding energy is comparable to that of other anticancer compounds, suggesting its potential as a lead compound for developing new inhibitors of various targets.

Curcumin has been discovered to effectively enhance cell proliferation in GBC-SD cells (gallbladder cancer) by increasing the number of cells in the S phase and decreasing the number of cells in the G0/G1 phase. Additionally, it triggered programmed cell death, reduced cell survival, initiated the cleavage of caspase-3, and controlled the ratio of Bcl-2 to Bax. Research has demonstrated that these effects are contingent upon the duration of exposure and the dosages of curcumin administered. Moreover, curcumin can hinder the process of DNA replication by obstructing the progression of S-phase cells, effectively restraining the growth of tumors [43]. Furthermore, curcumin increased the activity of mitogen-activated protein (MAP) kinase, diminished the functionality of Bcl-2, and reduced the levels of Bcl-2 phosphorylation (which is anti-apoptotic). Furthermore, it has been shown that curcumin did not affect the levels of NF-κB (a nuclear factor that prevents cell death) or Bax (a protein that promotes cell death) [44]. The expression of Bax/Bcl-2 was seen to exhibit a significant rise of 43% following curcumin therapy in the cell lines of mesothelioma, specifically MM-F1 and MM-B1.

In silico studies on EGCG, meaningful information about the molecular dynamics of the compound has been offered. Below are some of the findings; it has been found that EGCG has the ability to interact with different proteins, such as fibrous proteins, to cause disaggregation to block neurodegenerative diseases and amyloidosis [45]. Computational docking and X-ray crystallographic analyses have shown how this compound can form a complex with proteins and bind at or around the active site, thereby altering the conformation of the protein and subsequently inactivating its enzymatic and other biological functions. Ultimately, 12 signaling transduction pathways and 33 crucial target proteins have been explored using reverse docking and MD simulations on EGCG’s anti-tumor mechanisms. These investigations have also identified four potential EGCG target proteins linked to cancer development: IKBKB, KRAS, WEE1, and NTRK1. EGCG acts on specific proteins implicated in ailments such as cancers, metabolic syndrome, and inflammation that would otherwise lead to adverse health impacts. It was further found that the initial stage of aggregation of TDP-43 protein, which is involved in some proteinopathies such as ALS and FTLD, is effectively prevented by EGCG [45, 46]. These in silico studies have given substantial information regarding the molecular regulation of EGCG, which would help hunt for new therapeutic approaches to diverse diseases.

Epigallocatechin-3-gallate (EGCG) activated the intrinsic mitochondrial pathway, leading to the induction of apoptosis in multiple cancerous cells. This activation occurred through the activation of caspase 9 [47]. The induction of apoptosis in pancreatic cancer cells has been shown by the activation of the extrinsic death receptor pathway, specifically including the activation of Fas, DR5, and caspase 8 [48]. Moreover, EGCG demonstrated a decrease in the expression of anti-apoptotic proteins, specifically BCL-2, across multiple cancer cell lines. Furthermore, it was discovered that EGCG could stimulate death receptors, activate both caspase-dependent and caspase-independent pathways, and induce apoptosis in NCI-H295 human adrenal cancer cells [48]. This process was facilitated by controlling regulatory proteins and the induction of endoplasmic reticulum stress. Specifically, the administration of EGCG resulted in an upregulation of pro-apoptotic proteins BAD and BAX while concurrently downregulating the expression of anti-apoptotic proteins BCL-2, BCL-XL, and XIAP. The induction of apoptotic cell death in human glioma (U-87MG) cells was seen upon treatment with curcumin, which was found to modulate the activities of caspase 3 and Bcl-2. EGC activated Caspase-3 and PARP, down-regulated the phosphorylation of STAT3, and decreased Bcl-2 expression [49].

Furthermore, it effectively cleaved poly(ADP-ribose) polymerase (PARP) [50]. The promotion of apoptosis was facilitated by the overexpression of extracellular signal-regulated kinase 1/2 (ERK1/2) and the activation of caspase 9. Additionally, it was shown that curcumin promoted the nuclear translocation of p53. Furthermore, it exerted regulatory authority over the signaling cascades that relied on the nuclear factor (NF), facilitating the initiation of programmed cell death (apoptosis) [51].

The novelty of this research lies in its integration of traditional Chinese medicine (TCM) with advanced computational drug discovery techniques to address a critical challenge in cancer therapy: the suppression of Bcl-2 protein, widely known in cancer cell survival. The employment of pharmacophores modeling and molecular docking as methods of screening drugs from the database of TCM is a new approach that combines traditional and modern approaches. Identifying EGCG as the most powerful inhibitor of Bcl-2 function not only stresses the importance of natural products in anticancer therapy but also gives new directions for future research. This investigative work represents a groundbreaking effort to utilize in an organized way the vast and overlooked repository of TCM compounds to solve the problems of modern medicine, particularly in the field of oncology.

Despite some limitations, the study has a considerable contribution to the study of Bcl-2 inhibitors. On the one hand, the study has to emphasize the computational models and simulations strongly, but they can only render the complexity of biological systems to a limited extent. The assumptions on which ADMET analysis, pharmacophore modeling, and molecular docking are founded may not cover all human body processes, so these approaches could only be precursors to successful drug development. Additionally, the study concentrates on the EGCG and curcumin, which present beneficial features, but it ignores the synergistic action of the additional compounds. A pertinent limitation is the extrapolation factor between in vitro and computational findings when understanding how a drug works in a living system. This is always a challenge due to the metabolic and systemic variables not considered at the beginning of drug discovery.

The investigation has identified the limitations and thus provides a glimpse into the following research areas. One of the most essential steps will be to confirm the computational results both in vitro and in vivo by performing corresponding experiments. These studies would underpin the EGCG activity concerning the downregulation of Bcl-2 and evaluate its therapeutic efficacy and safety in biological systems. Additional investigation of the TCM compound database will likely reveal several other candidates and combinations of compounds with potential anti-cancer activity. Furthermore, looking into the molecular mechanisms for the effects made clear by the experiments will bring more to light about how these compounds bind with Bcl-2 and other associated proteins, thus unveiling a broader field of application. Besides the preclinical research involving pharmacokinetic and pharmacodynamic studies, further research should be expanded. This would help the compounds advance to clinical trials. Considering the possibility of leveraging these findings towards the personalization of medicine, bearing in mind the discrepancies in cancer’s genetics and molecular diversity might significantly contribute to the development of cancer-targeted therapies.

## Conclusions and future directions

The efficacy of polyphenols in cancer treatment has demonstrated promising outcomes in both in vitro and in vivo research. Various methodologies have been employed to investigate the antiproliferative effects of polyphenols, including the induction of cell cycle arrest, the decrease in levels of bcl-w and cyclin-CDK, and the increase in levels of p53. Polyphenols can modulate apoptotic mechanisms, including apoptosis, through their interaction with mitochondria and endoplasmic reticulum pathways. The interaction increases the Bax/Bcl-2 ratio and levels of reactive oxygen species (ROS), promoting the release of cytochrome c. Polyphenols employ receptor-mediated apoptosis as an alternative technique by activating several enzymes such as TNF-, caspase-8, and Bax oligomerization and relying on the mitochondrial apoptotic pathway. This study, in particular, finds its uniqueness in using a novel drug-discovery method that incorporates the old traditional medicine with the modern computational approaches aimed at the old, ever-challenging cancer therapy. Despite its limitations and well-known issues of initial studies, this research outlines how future scaling-up research can be implemented. Through combined efforts of multiple disciplines, new action mechanisms will be discovered, and the pharmacological potential of TCM and the possible anti-cancer use of compounds like EGCG will be elucidated.

### Electronic supplementary material

Below is the link to the electronic supplementary material.

Supplementary Material 1

## Data Availability

All the data is presented in the manuscript and is available with the online version of the manuscript.
