# Supplementary material for: Epigallocatechin gallate and curcumin inhibit Bcl-2: a pharmacophore and docking based approach against cancer
Source: Breast Cancer Res. 2024 Jul 8;26:114. doi: 10.1186/s13058-024-01868-9 (PMC11229278; doi:10.1186/s13058-024-01868-9)
Supplement: Supplementary file 1 — Supplementary Material 1 [file 13058_2024_1868_MOESM1_ESM.docx]

**Table S1**. Active and Inactive compounds

| **Active** | **Inactive** |
| --- | --- |
| Resveratrol | Apigenin |
| Curcumin | Catechins |
| Epigallocatechin gallate (EGCG) | Caffeic acid |
| Quercetin | Gallic acid |
| Kaempferol | Chlorogenic acid |
| Ellagic acid | Ferulic acid |
| Rutin | Rosmarinic acid |
| Myricetin | Hesperidin |
|  | Silibinin |
|  | Oleuropein |
|  | ECG (Epicatechin gallate) |
|  | Procyanidins |
|  | Piceatannol |
|  | Gallocatechin gallate |
|  | Isoflavones |
|  | Phloretin |
|  | Curcuminoids |
|  | Cyanidin |
|  | Delphinidin |
|  | Peonidin |
|  | Malvidin |
|  | Pelargonidin |
|  | Theaflavins |
|  | Thearubigins |
|  | Hesperetin |
|  | Nobiletin |
|  | Tangeretin |
|  | Isorhamnetin |
|  | Morin |
|  | Genipin |
|  | Quercetagetin |
|  | Tyrosol |
|  | Vanillic acid |
|  | Syringic acid |
|  | Lycopene |
|  | Genistein |
|  | Luteolin |
|  | Naringenin |

**Table S2. Active sites identified with the Sitemap tool**

| **Title** | **Site Score** | **residues** |
| --- | --- | --- |
| sitemap_6O0K_site_1 | 0.933 | Chain A: 104,108,111,112,114,115,133,136,137,149,150,152,153,156 |
| sitemap_6O0K_site_2 | 0.732 | Chain A: 12,15,16,30,32,33,171,174,175,178 |
| sitemap_6O0K_site_5 | 0.699 | Chain A: 96,99,100,103,104,107,108,144,145,148,198,202,203 |
| sitemap_6O0K_site_4 | 0.623 | Chain A: 127,130,131,134,135,176,179,180,184 |
| sitemap_6O0K_site_3 | 0.617 | Chain A: 9,10,11,12,14,181,186,189,190,194,195 |
